# The link between Pos-Traumatic Stress Disorder and Childbirth

**DOI:** 10.1192/j.eurpsy.2023.2390

**Published:** 2023-07-19

**Authors:** A. M. Fraga, A. Quintão, B. Mesquita, C. Melo Santos, F. Soares, J. Correia, M. Albuquerque, S. Neves, A. Moutinho, P. Cintra

**Affiliations:** 1 Departamento de Psiquiatria e Saúde Mental, Hospital de Cascais, Cascais; 2 Serviço de Psiquiatria, Centro Hospitalar Lisboa Ocidental, Lisboa; 3 Serviço de Psiquiatria, Unidade de Saúde Local do Nordeste, Bragança; 4 Serviço de Psiquiatria, Centro Hospitalar de Tâmega e Sousa, Penafiel, Portugal

## Abstract

**Introduction:**

Childbirth can be experienced as distressing or even traumatic for some women and her partners, which could cause psychological distress, intense fear or helplessness and increases the risk of anxiety, depression and even post-traumatic stress disorder (PTSD). The reported prevalence of post-traumatic stress disorder after childbirth ranges from 1.5% to 6%.

**Objectives:**

The current study aimed to elaborate a narrative literature review to identify predictors associated development of PTSD in women and the partners.

**Methods:**

PubMed database searched using the terms “post-traumatic stress disorder” and “childbirth” and “trauma”. Only research conducted in the past 20 years was considered for inclusion.

**Results:**

Several variables were associated with risk to development PTSD after childbirth, including negative experiences and severe fear of childbirth, subjetive distress, previous abortion, psychological difficulties in pregnancy, previous psychiatric problems, history of PTSD and trauma. Futhermore, obstretic and birth-related factors such as pregnancy complications, type of birth could also contribute to PTSD in women and her partners. Additionally, diferent environmental factors like poor interaction between provider and mother, low social support during labour and birth are associated with development of PTSD.

**Conclusions:**

Clinicians should be aware that many women and her parterns have a risk to development PTSD following childbirth. We need to research risk factors in routine clinical practice and carefully monitored the patients with high risk.

**Disclosure of Interest:**

None Declared

